# Extensive Deep Vein Thrombosis and Pulmonary Embolism in a Young Patient With Heterozygous Factor V Leiden Mutation and Antiphospholipid Syndrome

**DOI:** 10.7759/cureus.72425

**Published:** 2024-10-26

**Authors:** Elena A Sava, Claudiu I Sântean, Andrei Manea, Nicoleta M Crăciun-Ciorba

**Affiliations:** 1 Cardiology, Emergency Institute for Cardiovascular Diseases and Transplantation, Targu Mures, ROU; 2 Radiology, County Emergency Clinical Hospital of Targu Mures, Targu Mures, ROU; 3 Internal Medicine, County Emergency Clinical Hospital of Targu Mures, Targu Mures, ROU; 4 Internal Medicine, University of Medicine, Pharmacy, Science, and Technology of Targu Mures, Targu Mures, ROU

**Keywords:** anticardiolipin antibodies, anticoagulant treatment, antiphospholipid syndrome, deep vein thrombosis, factor v leiden mutation, pulmonary embolism

## Abstract

Although thrombotic events are uncommon in young individuals, patients with genetic mutations in coagulation factors may develop extensive multisite thrombosis. We present the case of a 26-year-old patient, a smoker for nine years, who was admitted to the hospital complaining of right thigh pain with swelling, right flank abdominal pain, dyspnea, and hemoptysis. A medical history provided by the patient indicated that one month prior to presentation, an accidental fall had resulted in multiple rib fractures, bilateral hemopneumothorax, and pneumomediastinum. These injuries were treated with bilateral pleurotomy and passive pleural drainage. Upon initial presentation, the electrocardiogram (ECG) exhibited a distinctive pattern indicative of pulmonary embolism (PE), specifically sinus tachycardia and the S1Q3T3 pattern. Additionally, the thoraco-abdominopelvic angio-CT revealed acute PE in the branches of the pulmonary arteries supplying the bilateral lower lobes and deep vein thrombosis in the right external iliac vein, right common iliac vein with a protruding filling defect of approximately 18 mm in the inferior vena cava and extension to the left common iliac vein. Furthermore, a Doppler ultrasound of the right lower extremity revealed the presence of thrombosis along the entire right femoro-popliteal axis. Subsequent screening for hereditary thrombophilia revealed a heterozygous factor V Leiden G1691A* mutation and elevated levels of anticardiolipin antibodies. Despite the patient's compliance with non-vitamin K antagonist oral anticoagulants (NOACs) following discharge, a new thrombotic event, left femoral-popliteal venous axis thrombosis, occurred after a three-month interval. Repeat testing for anticardiolipin antibodies revealed an elevated titer, leading to a diagnosis of antiphospholipid syndrome. Given this diagnosis, anticoagulation with an antivitamin K was initiated. Screening for hereditary thrombophilia is crucial in young patients with thromboembolic events, as genetic causes may influence the therapeutic management and duration of anticoagulant treatment.

## Introduction

Venous thromboembolism (VTE) is a clinical entity that encompasses both deep vein thrombosis (DVT) and pulmonary embolism (PE). The pathophysiology of VTE has been extensively investigated by Rudolf Virchow. He delineated three categories of factors that contribute to thrombosis: stasis of blood flow, endothelial injury, and hypercoagulability. Circulatory stasis is frequently the result of prolonged periods of immobilization, venous obstruction, congenital abnormalities of the venous circulation, and heart or lung failure. Endothelial injury is closely related to vascular wall trauma, venous punctures, the use of venous catheters, thrombophlebitis, cellulitis, and atherosclerosis. The condition of hypercoagulability may be precipitated by a number of factors, including major surgery, trauma, pregnancy, sepsis, inflammatory syndromes (such as inflammatory bowel disease, systemic lupus erythematosus, and antiphospholipid syndrome (APS)), dehydration, estrogen therapy, and inherited thrombophilia (such as factor V Leiden, prothrombin mutation, protein C, protein S, and antithrombin deficiency) [1].

The factor V Leiden G1691A mutation represents one of the most prevalent causes of inherited thrombophilia. Factor V Leiden is an abnormal variant of factor V that becomes resistant to the action of activated protein C. This genetic defect is caused by a point mutation in the gene encoding factor V synthesis on chromosome 1q23, resulting in the substitution of guanine for adenine at position 1691, which leads to the substitution of arginine with glutamine at amino acid 506. This mutation predisposes patients to thrombotic events because activated factor V (FVa) is unable to undergo cleavage by activated protein C due to the absence of arginine 506, which is the primary cleavage site [2]. It has been estimated that 5% of patients with the heterozygous factor V Leiden mutation will have developed thromboembolic events by the age of 65 years.

APS is an autoimmune inflammatory disorder characterized by the presence of persistent antibodies that predispose to thrombotic events and pregnancy loss. These antibodies, which are directed against phospholipids, cause an increased risk of blood clots in blood vessels. The antibodies in question include anti-cardiolipin IgG or IgM antibodies, anti-beta-2-glycoprotein-1 IgG or IgM antibodies, and lupus anticoagulant antibodies. These antibodies bind to membrane phospholipid-binding proteins, thereby triggering the activation of endothelium, platelets, and leukocytes, which in consequence leads to in situ thrombosis [3].

## Case presentation

**Figure 1:**
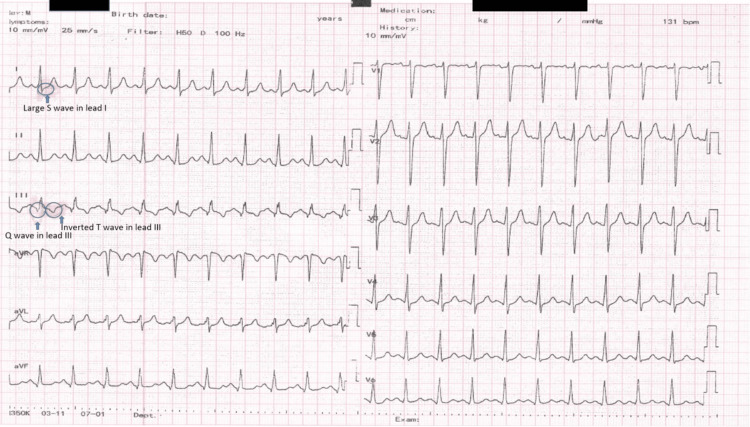
ECG: Sinus tachycardia, heart rate 131 bpm, intermediate QRS axis, PR duration 140 ms, QRS duration 100 ms, S1Q3T3 pattern ECG: electrocardiogram

A 26-year-old male patient with a nine-year history of smoking was admitted to the hospital complaining of right thigh pain and swelling, right flank abdominal pain, dyspnea, and hemoptysis. The patient had a history of an accidental fall from a height of three meters one month prior to presentation, resulting in multiple rib fractures, a comminuted fracture of the left clavicle, bilateral hemopneumothorax, and pneumomediastinum. These injuries were treated with bilateral pleurotomy and Beclere passive pleural drainage. The patient was monitored dynamically with serial chest X-rays, which demonstrated a favorable evolution with resolution of the hemopneumothorax and re-expansion of both lungs. Upon discharge from the thoracic surgery department, the attending physician prescribed prophylactic antibiotics and antifungal treatment. To prevent bacterial superinfection, the surgeon's choice was oral Cefuroxime. This was administered concomitantly with antifungal treatment to prevent a possible fungal infection that could have occurred following the destruction of the normal bacterial flora by the antibiotic treatment. At home, the patient underwent oral antibiotic treatment with Cefuroxime 500 mg twice daily, prophylactic antifungal treatment with Fluconazole 150 mg every three days, probiotics, and analgesics. Furthermore, the patient had been confined to bed rest for the majority of the previous month, and the initiation of postoperative anticoagulant prophylaxis was not feasible due to the presence of bilateral hemopneumothorax. Upon admission, the patient exhibited a stable hemodynamic and respiratory status with an oxygen saturation of 99% on room air, no pulmonary rales, a heart rate of 90 beats per minute, a blood pressure of 120/80 millimeters of mercury, and no murmurs. Additionally, the patient presented with severe right thigh pain and edema, as well as right lower quadrant abdominal pain. Laboratory tests revealed an elevated D-dimer level, leukocytosis with neutrophilia, thrombocytosis, and normal renal function (Table 1). The electrocardiogram (ECG) displayed a pattern suggestive of a PE, exhibiting sinus tachycardia and the S1Q3T3 pattern (Figure 1).

**Table 1:** Laboratory tests

Laboratory test	Result	Reference range
D-Dimer	>5 μg/mL	<0.5 μg/mL
White blood cell count	16.8×10^3^ /μL	3.6-10×10^3^ /μL
Neutrophils	13.6×10^3^ /μL	1.4-6.5×10^3^ /μL
Platelet count	496×10^3^ /μL	150-450×10^3^ /μL
Creatinine	0.83 mg/dL	0.72-1.25 mg/dL
Urea	25 mg/dL	19.04-44.08 mg/dL

A transthoracic echocardiogram revealed a non-dilated left ventricle with preserved systolic function, a left ventricular ejection fraction (LVEF) of 55%, a non-dilated right ventricle, a tricuspid annular plane systolic excursion (TAPSE) of 18 mm, no significant hemodynamic valvulopathies, an inferior vena cava (IVC) of 14 mm with inspiratory collapse >50%, and a non-dilated ascending aorta without dissection flap.

**Figure 2:**
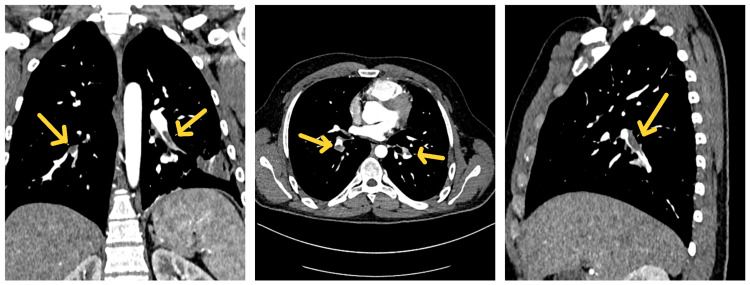
Acute pulmonary embolism in the branches of the pulmonary arteries supplying the bilateral lower lobes

**Figure 3:**
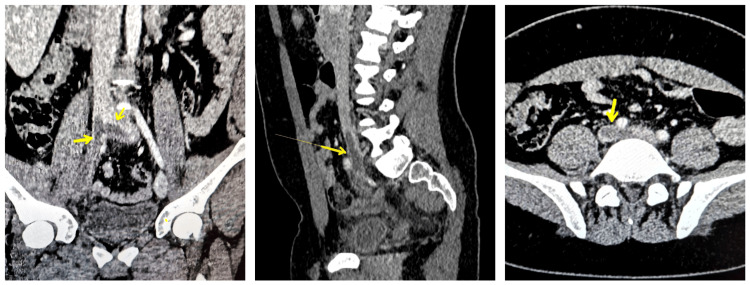
Deep vein thrombosis in the right external iliac vein, right common iliac vein, with a protruding filling defect of approximately 18 mm in the inferior vena cava, extending into the left common iliac vein

A thoraco-abdominopelvic angio-CT revealed the presence of acute PE in the branches of the pulmonary arteries supplying the bilateral lower lobes (Figure 2) and DVT in the right external iliac vein, right common iliac vein, with a protruding filling defect of approximately 18 mm in the IVC extending into the left common iliac vein (Figure 3).

In light of these findings, treatment with unfractionated heparin was initiated in the emergency department. This consisted of a 5000 IU bolus followed by an intravenous infusion at 18 IU/kg/min, with subsequent dose adjustment based on activated partial thromboplastin time (APTT). During the course of hospitalization, the patient received hydration, a proton pump inhibitor (40 mg of Pantoprazole once daily), antibiotic therapy with Ceftriaxone (1 g twice daily), probiotics, and was switched to low molecular weight heparin (LMWH) (Enoxaparin 2x0.8 ml).

**Figure 4:**
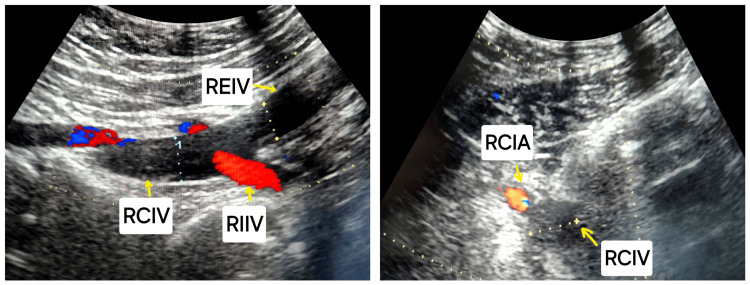
Doppler ultrasound revealing the absence of blood flow from RCIV and REIV RCIV: right common iliac vein; REIV: right external iliac vein; RIIV: right internal iliac vein; RCIA: right common iliac artery

A venous Doppler ultrasound (Figure 4) was performed, which showed a lack of complete compressibility and an absence of blood flow in the right femoro-popliteal axis, right external iliac vein, right common iliac vein extending to the IVC, and left common iliac vein.

**Figure 5:**
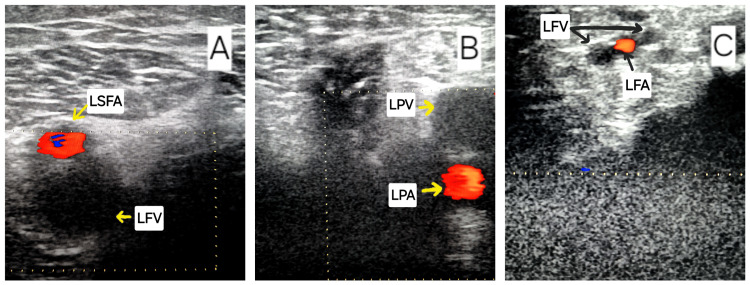
A) Femoral vein thrombosis; B) Popliteal vein thrombosis; and C) Fibular vein thrombosis A) LSFA: left superficial femoral artery; LFV: left femoral vein B) LPV: left popliteal vein; LPA: left popliteal artery C) LFV: left fibular veins; LFA: left fibular artery

In the context of the thrombotic events observed in a young male patient with transient risk factors (chest trauma, smoking, and immobilization), the possibility of a genetic predisposition to thrombotic events was considered, and screening for inherited thrombophilia was conducted. The screening results indicated the presence of a heterozygous factor V Leiden G1691A mutation and an anticardiolipin antibody level of 55 mg/dL (normal range: <15 mg/dL). Less than 48 hours after admission, the patient presented fever and dry cough. Consequently, rapid tests for SARS-CoV-2 and influenza A and B were conducted, yielding negative results. Additionally, blood and urine cultures were obtained, yielding negative results. The antibiotic regimen was modified to include Moxifloxacin 400 mg once daily. After five days of treatment with Moxifloxacin and Ceftriaxone, the patient remained febrile with hemodynamic and respiratory instability, a blood pressure of 80/50 mmHg requiring norepinephrine, and oxygen saturation of 80% in room air requiring oxygen therapy, leading to the decision to repeat the contrast-enhanced thoracic CT scan, which showed a right infected lung infarct and persistent filling defects in the pulmonary circulation. Consequently, the antibiotic treatment was modified to include Meropenem 2 g three times a day. The patient's condition improved, with no signs of respiratory distress or fever. The pain in the left lower extremity also diminished, and laboratory tests indicated a reduction in inflammatory markers. A venous Doppler ultrasound was conducted prior to the patient's discharge, which revealed complete recanalization of the IVC and partial recanalization of the other affected veins in comparison to the previous examination. The patient was discharged after a hospital stay of two weeks, with instructions to continue anticoagulant treatment at home (Apixaban 10 mg twice daily for one week, then 5 mg twice daily). A subsequent examination three months later revealed the occurrence of a new thrombotic event, namely left femoro-popliteal axis thrombosis and left fibular vein thrombosis (Figure 5).

Furthermore, the anticardiolipin antibody level remained elevated at 63 mg/dL on repeat assessment, thereby confirming the presence of APS. Consequently, the non-vitamin K antagonist (VKA) oral anticoagulant (NOAC) treatment was discontinued, and Acenocoumarol was initiated at a dosage level adjusted to maintain an international normalized ratio (INR) between 2 and 3.

## Discussion

VTE is a relatively uncommon occurrence in the younger demographic. In this case study, we present the case of a young male patient with multiple concomitant risk factors, including smoking, recent major chest trauma, lack of postoperative thromboprophylactic anticoagulation, and prolonged immobilization. Although these risk factors are transient, we consider screening for hereditary thrombophilia to be an appropriate course of action. Therefore, in addition to the aforementioned transient risk factors, we initially diagnosed the coexistence of a new, permanent risk factor: a heterozygous factor V Leiden mutation.

Imaging played an indispensable role in the diagnosis of PE and DVT, which was straightforwardly established by angio-CT. However, the selection of the most appropriate treatment and its duration presented a multitude of challenges. While anticoagulant therapy represents the cornerstone of treatment for these patients, it is essential to weigh the potential complications that may arise. In the case of our patient, the initial anticoagulant treatment consisted of unfractionated heparin. Despite the patient having received unfractionated heparin (UFH) in the emergency department, we opted to switch to LMWH to minimize the risk of potential heparin-induced thrombocytopenia (HIT) [4] and for the convenience of the patient, eliminating the need to measure the APTT level every six hours.

Another challenge was determining the most appropriate oral anticoagulant for chronic home treatment. Given the lack of consensus on the optimal anticoagulant for patients with a heterozygous factor V Leiden mutation, we sought to analyze similar cases or studies involving small patient groups to identify the most effective anticoagulant. Direct oral anticoagulants (DOACs) have been demonstrated to be non-inferior to VKAs for the management of DVT/acute PE. With a reduced risk of bleeding and the absence of the need for international normalized ratio (INR) monitoring, a DOAC was selected for our patient [5]. Studies found that patients with protein S deficiency were successfully treated with edoxaban [6,7] or rivaroxaban [8,9], protein C deficiency with rivaroxaban [10,11] or edoxaban [12,13], antithrombin deficiency with apixaban [14] or rivaroxaban [15], homozygous factor V Leiden mutation with rivaroxaban [16], and heterozygous factor V Leiden mutation with apixaban [17].

The START registry [18], a multicenter cohort study, included 446 patients with thrombophilia, comprising 22 with antithrombin deficiency, 28 with protein C deficiency, 41 with protein S deficiency, 101 with heterozygous or homozygous F2C*97G>A mutation, 205 with heterozygous or homozygous FVL mutation, and 49 with combined thrombophilia. Of these, 172 were treated with VKAs and 274 with DOACs. Patients treated with NOACs experienced a significantly lower incidence of non-major hemorrhagic events (p=0.01) compared to those on VKAs. However, there was no statistically significant difference in major hemorrhagic events or thrombotic events between the two groups.

An Italian cohort study [19] assessed the safety and efficacy of novel oral anticoagulants in 45 patients with severe thrombophilia. Of the cohort, 33 patients switched from VKA therapy to novel oral anticoagulants due to fluctuations in international normalized ratio (INR) values and the difficulty of maintaining regular INR monitoring. The mean duration of VKA therapy was 60 months, and the follow-up period after initiating NOACs was 29 months. No patients experienced thrombotic or hemorrhagic events while on NOACs, whereas three minor hemorrhagic events and two thrombotic events occurred during VKA treatment.

In consideration of the aforementioned data, at the time of discharge, we determined that the optimal anticoagulant for our patient is Apixaban at a dose of 10 mg twice a day for seven days, followed by 5 mg twice a day. Additionally, determining the optimal duration of anticoagulant therapy presented a significant challenge. A review of the literature on the duration of anticoagulant therapy for patients with a heterozygous factor V Leiden mutation at the first thromboembolic event indicates that the recommended treatment period should be the same as for patients without genetic mutations. In cases where other risk factors are present, the treatment duration may be extended beyond the typical 3-6 months if there are additional major temporary or permanent risk factors. Some literature indicates that long-term anticoagulation may be beneficial for patients with a heterozygous factor V Leiden mutation who experience idiopathic thromboembolic events, particularly in those with a high risk of recurrence and a low hemorrhagic risk. In light of the patient's age (26 years), smoking history, recent major thoracic trauma, and the presence of multiple thrombosis sites (pulmonary, lower extremities, right external iliac vein, right and left common iliac veins, and IVC) in the absence of other hemorrhagic risk factors, we determined that the patient requires long-term anticoagulant therapy.

Another distinctive feature of this case is the presence of elevated anticardiolipin antibodies, which raised our suspicion of APS. At the three-month follow-up, despite the patient's compliance with NOAC anticoagulation following discharge, he presented with a new thrombotic event, specifically left femoral-popliteal venous axis thrombosis. The cardiolipin antibody titer was repeated and remained elevated, confirming the diagnosis of APS. Many clinical trials have shown an increased risk of recurrent thrombotic events associated with NOACs compared with VKA in patients with APS. In 2021, a review [20] was conducted on a limited number of patients to evaluate the efficacy of NOACs in the prevention of thrombotic events in patients with APS. The findings indicate that patients with arterial APS or triple positivity (anti-cardiolipin (aCL), anti-β2 glycoprotein I (β2GPI), and lupus anticoagulant (LAC)) should be treated with VKA, whereas those with venous APS and single or double positivity may be candidates for NOACs. However, further high-quality studies are required to confirm this. In the case of our patient, despite the diagnosis of venous APS and single positivity, a new thrombotic event occurred following initial treatment with Apixaban. Consequently, a switch to a VKA was deemed appropriate. So we stopped the NOAC and started Acenocoumarol with doses adjusted to maintain an INR between 2 and 3.

## Conclusions

Notwithstanding the presence of multiple transient risk factors for VTE (including thoracic trauma from a three-meter fall, bilateral hemopneumothorax requiring drainage, and delayed postoperative thromboprophylaxis, in addition to bed rest and smoking), the necessity for screening for hereditary thrombophilia persists, given the existence of an extensive deep venous thrombosis associated with PE. The identification of a heterozygous factor V Leiden mutation and the diagnosis of APS prompted a change in the therapeutic approach to long-term anticoagulation for this patient.

Although NOACs are the preferred anticoagulant for VTE associated with factor V Leiden mutation, the superimposition of APS requires a different therapeutic approach. In a patient with a genetic mutation who is adhering to a properly prescribed NOAC anticoagulant regimen, a recurrent cause of thrombotic events may be an undiagnosed APS. Given the lack of studies demonstrating the efficacy of NOAC anticoagulation in APS, current guidelines recommend oral anticoagulation with anti-vitamin K.

## References

[1] Wolberg AS, Aleman MM, Leiderman K, Machlus KR (2012). Procoagulant activity in hemostasis and thrombosis: Virchow's triad revisited. Anesth Analg.

[2] Segers O, Castoldi E (2009). Factor V Leiden and activated protein C resistance. Adv Clin Chem.

[3] Petri M (2020). Antiphospholipid syndrome. Transl Res.

[4] Bautista Sanchez R, Gely Y, Iglesias JN (2022). Homozygous Factor V Leiden complicated by heparin-induced thrombocytopenia: a case report. J Med Cases.

[5] van Es N, Coppens M, Schulman S, Middeldorp S, Büller HR (2014). Direct oral anticoagulants compared with vitamin K antagonists for acute venous thromboembolism: evidence from phase 3 trials. Blood.

[6] Lee WC, Huang MP (2021). Lead thrombus under standard-dose edoxaban in a patient with normal to high creatinine clearance and protein S deficiency. Thromb J.

[7] Yagi S, Kagawa K, Fujimoto E, Sata M (2019). Recurrent venous thrombosis during direct oral anticoagulant therapy in a patient with protein S deficiency. J Med Invest.

[8] Wang T, Zhao XJ, Zhu HD, Lu M, Wen B, Ma L (2021). Clinical characteristics, genes identification and follow-up study of a patient with central venous thrombosis from a protein S deficiency pedigree. Eur Rev Med Pharmacol Sci.

[9] Krumb E, Hermans C (2020). An exceptional case of severe combined inherited thrombophilia successfully treated with rivaroxaban. Blood Coagul Fibrinolysis.

[10] Sun L, Li X, Li Q, Wang L, Li J, Shu C (2021). Multiple arterial and venous thromboembolism in a male patient with hereditary protein C deficiency: a case report. Medicine (Baltimore).

[11] Menon N, Sarode R, Zia A (2018). Rivaroxaban dose adjustment using thrombin generation in severe congenital protein C deficiency and warfarin-induced skin necrosis. Blood Adv.

[12] Saito K, Ishii K, Furuta K, Kobayashi M, Wada Y, Morishita E (2021). Recurrent cerebral venous thrombosis treated with direct oral anticoagulants in a Japanese man with hereditary protein C deficiency. J Stroke Cerebrovasc Dis.

[13] Watanabe K, Arakawa Y, Yanagi M, Isobe K, Mori M, Koh K (2019). Management of severe congenital protein C deficiency with a direct oral anticoagulant, edoxaban: a case report. Pediatr Blood Cancer.

[14] Nojima Y, Ihara M, Adachi H, Kurimoto T, Nanto S (2019). Efficacy and safety of apixaban in a patient with systemic venous thromboembolism associated with hereditary antithrombin deficiency. J Cardiol Cases.

[15] Minami K, Kumagai K, Sugai Y, Nakamura K, Naito S, Oshima S (2018). Efficacy of oral factor Xa inhibitor for venous thromboembolism in a patient with antithrombin deficiency. Intern Med.

[16] Cook RM, Rondina MT, Horton DJ (2014). Rivaroxaban for the long-term treatment of spontaneous ovarian vein thrombosis caused by factor V Leiden homozygosity. Ann Pharmacother.

[17] Costa RL, Triggs M, Cole SE, Lacey J, Reddy S (2020). Anticoagulation therapy in a patient with heterozygous factor V Leiden and coexisting homozygous prothrombin gene mutations. Cureus.

[18] Margaglione M, Antonucci E, D'Andrea G (2020). Anticoagulation in Italian patients with venous thromboembolism and thrombophilic alterations: findings from START2 Register study. Blood Transfus.

[19] Serrao A, Lucani B, Mansour D (2019). Direct oral anticoagulants in patients affected by major congenital thrombophilia. Mediterr J Hematol Infect Dis.

[20] Pastori D, Menichelli D, Cammisotto V, Pignatelli P (2021). Use of direct oral anticoagulants in patients with antiphospholipid syndrome: a systematic review and comparison of the international guidelines. Front Cardiovasc Med.

